# Recurrence Rate in a Patient Treated with Colon Resection Followed by Chemotherapy in Comparison to a Patient Treated with Colon Resection without Chemotherapy

**DOI:** 10.7759/cureus.7544

**Published:** 2020-04-04

**Authors:** Khalid A Alshehri, Talal M Altuwaylie, Abdulaziz Fakieha, Ghassan AlGhamdi, Saad M Alshahrani, Zaher Mikwar

**Affiliations:** 1 Medicine, College of Medicine, King Saud Bin Abdulaziz University for Health Sciences, Jeddah, SAU; 2 Surgical Oncology, King Abdulaziz Medical City, Ministry of National Guard Health Affairs, Jeddah, SAU

**Keywords:** colon cancer recurrence, general surgery, colon cancer, jeddah, chemotherapy, without chemotherapy, recurrence rate, colon resection, national guard health affairs (ngha), kingdom of saudi arabia (ksa)

## Abstract

Given that colon cancer is one of the most prevalent cancers worldwide, it is essential to employ strategies to try to reduce its incidence and recurrence rate. Though colon cancer is a sporadic disease in the vast majority of cases, multiple risk factors are linked to this disease, namely, obesity and cigarette smoking. Additionally, not many studies have been done in Saudi Arabia studying the recurrence rate of colon cancer. Therefore, we conducted a retrospective cohort study at King Khalid Hospital, King Abdulaziz Medical City, National Guard Health Affairs (NGHA), Jeddah, Saudi Arabia to investigate the recurrence rate of colon cancer in patients treated with complete colon resection followed by chemotherapy versus patients treated with colon resection alone via electronic and paper medical records. A total of 120 patients were included in this study; 61 were males (50.8%) and 59 were females (49.2%). According to our findings, the recurrence rate in patients who underwent surgical resection with adjuvant chemotherapy was 15.6% (n = 10), while the recurrence rate in patients with surgery alone was 21.4% (n = 12). Cancer recurrence is associated with significant morbidity and mortality. Therefore, further studies should be done to investigate the recurrence rate in patients with risk factors to identify and deal with the causes of recurrence.

## Introduction

Colon cancer is a cancerous tumor arising from the inner wall of the large intestine (colon). It starts as noncancerous small clusters of cells called polyps on the inside wall of the colon [1]. Without treatment, some of the benign polyps can become cancerous. It is the most common type of gastrointestinal tumors worldwide. In Saudi Arabia, it is the most common cancer among men and the third among women [2]. Although it can happen at any age, it usually affects older adults (above 50). Many factors may increase the risk of colon cancer, such as older age (over 50), African-American race, a personal or family history of colon cancer or polyps, chronic inflammatory diseases of the colon, such as ulcerative colitis and Crohn’s disease, low-fiber or high-fat diet, smoking, alcoholism, and obesity [3].

Symptoms of colon cancer depend on the stage. Many people with colon cancer experience no symptoms in the early stages of the disease. Therefore, regular screening is important and should start at age 50. When symptoms appear, they'll likely vary, depending on the size and location of the cancer in the large intestine. Generally, signs and symptoms of colon cancer include a persistent change in the bowel habits, including diarrhea, constipation, or a change in stool consistency, rectal bleeding, persistent abdominal discomfort (such as cramps, gas or pain), a feeling that the bowel does not empty, weakness or fatigue, and unexplained weight loss [4].

The American Joint Committee on Cancer (AJCC) categorizes colon cancer patients based on the extent of the primary tumor (T), lymph node involvement (N), and the presence of metastasis (M) [5]. The tumor node metastasis (TNM) staging system is used to stratify colon cancer into five main stages (0 to IV) to facilitate and personalize the treatment of modality and prognosis. Stage 0 denotes carcinoma in situ where abnormal cells are confined to the colon mucosa and have a chance of becoming cancerous. In Stage 1 colon cancer, cancerous cells are found in the mucosa and submucosal layers, whereas in Stage II, cancerous cells invade either the outermost layer (IIA), spread to the visceral peritoneum (IIB), or other nearby organs (IIC). Moreover, lymph node involvement marks the beginning of Stage III that is further subdivided into three substages based on the number of lymph nodes involved or nearby tissue. In the final stage (Stage IV), colon cancer spreads to distant sites or organs, such as the lung, liver, ovary, or distant lymph node, and is subdivided into three groups based on the number of affected organs [6]. The AJCC staging of colon cancer has guided multidisciplinary teams of health providers in selecting the most appropriate intervention for each patient.

The most vital prognostic indicator of survival in colon cancer is the stage of the tumor established by TNM classification which governs the type of clinical treatment selected for cancer patients [7]. Surgical resection of colon cancer is the universal norm of treatment, especially for Stage 0 and I tumors, and laparoscopic surgery was introduced in the late nineties only to prove its commensuration, if not superiority, to open colectomy. A complete or partial colectomy is used in all five stages of colon cancer depending on the feasibility of resection and tissue involvement. A recent systematic review reported laparoscopic colectomy had lower risks of adhesion-related small bowel obstruction and incisional hernia; however, the three-year disease-free survival between open and laparoscopic surgeries remained unchanged [8].

The controversy arises when adjuvant chemotherapy is considered for Stage II colon cancer patients [9]. The National Comprehensive Cancer Network (NCCN) Guidelines for colon cancer do not clarify who might benefit from adjuvant chemotherapy. Nevertheless, the NCCN guidelines advise that adjuvant chemotherapy should be considered for Stage II patients with T4 staging, poorly differentiated tumors, lymphovascular or perineural invasion, obstructing or perforating cancers, positive margins, and unsatisfactory examined lymph nodes [9]. Traditionally used adjuvant chemotherapy regimens for Stage III colon cancer are either the FOLFOX (5-Fluorouracil, leucovorin, and oxaliplatin) or CapeOx (capecitabine and oxaliplatin) [7]. Several randomized studies conducted in the United States showed reductions in mortality and recurrence in patients who received adjuvant chemotherapy compared with those who underwent surgery alone, 33% and 40%, respectively. Furthermore, a large, population-based study conducted in the United States reported Stage III colon cancer patients on adjuvant chemotherapy initiated within three months had a 50% increase in colon cancer-specific mortality compared with initiating chemotherapy within one month [10].

Ejaz et al. stated that patients receiving chemotherapy had a better recurrence-free survival [11]. Another study in the Netherlands by van Erning et al. found out that adjuvant chemotherapy reduced recurrence in patients with Stage III colon cancer aged 75 years old or older [12]. Also, Kannarkatt et al. stated that the use of adjuvant chemotherapy in Stage III colon cancer had a relative risk reduction of 30% in cancer recurrence [13].

## Materials and methods

This retrospective cohort study was conducted at King Abdulaziz Medical City, National Guard Health Affairs (NGHA), Jeddah, Saudi Arabia. The study included adult patients 30 years old or older who were diagnosed with colon cancer and underwent surgical resection with or without adjuvant chemotherapy from 2009 to 2018. Patients were excluded if they received neoadjuvant chemotherapy.

The study data were collected using a data collection sheet consisting of five parts. The first part collected demographic characteristics and the second part collected risk factors associated with colon cancer. The third and fourth parts included the stages of colon cancer and treatment. The fifth part involved the recurrence of colon cancer. Patient names were not collected to maintain confidentiality. This questionnaire was filled by investigators. The questionnaire was not shared with the office staff or physician.

Data were analyzed using IBM SPSS Statistics for Windows, Version 20.0 (IBM SPSS Statistics, Armonk, NY). Participants were included using a non-probability convenience sampling technique, and the sample size was calculated using Rao online software (Raosoft, Inc., Seattle, WA). The margin of error was 5% with a 95% confidence level. This study was approved by our institutional review board.

## Results

**Figure 1:**
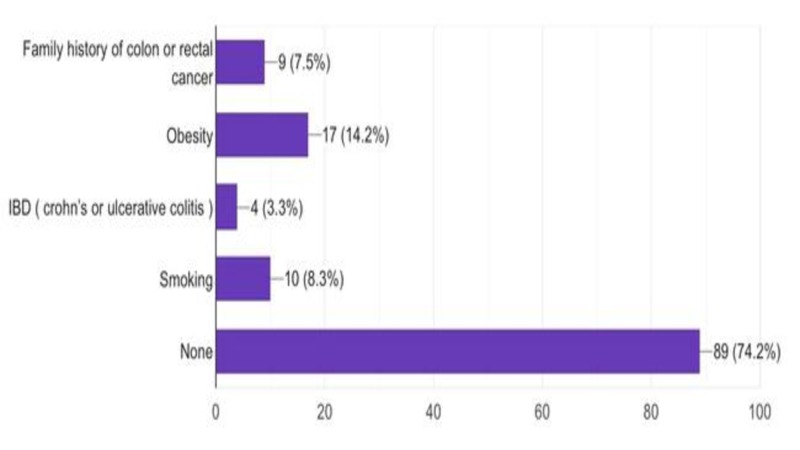
Risk factors associated with colon cancer

The study included 120 patients with colon cancer (61 males, 50.8%; 59 females, 49.2%). The mean age was 61 ± 13 years. The most common risk factor related to colon cancer in this research was obesity (n = 17; 14.1%), followed by smoking (n = 10; 8.3%) (Figure 1).

The most common site of colon cancer in this study was sigmoid (n = 50; 41.7%), followed by right colon cancer (n = 36; 30%), left colon cancer (n = 16; 13.3%), others (n = 14; 11.6%), and transverse colon cancer (n = 4; 3.3%). A total of 39 patients (32.5%) were diagnosed with Stage II colon cancer, 36 patient (30%) were diagnosed with Stage III, Stage IV colon cancer was found in 25 patients (20.8%), and 20 patients (16.7%) were diagnosed with Stage I. 

We found that 64 patients (53.3%) underwent surgical resection followed by chemotherapy, while 56 patients (46.7%) underwent surgery alone. The recurrence rate in patients who underwent surgical resection with adjuvant chemotherapy was (n = 10; 15.6%), while the recurrence rate in patients who underwent surgery alone was (n = 12; 21.4%). 

## Discussion

Globally, an estimated 1.48 million new cases occurred in 2012, ranking colon cancer the third most common cancer in the world [14]. According to the Saudi Cancer Registry, a total of 859 cases of colon cancer were diagnosed in 2015, making it the second most common cancer. In our study, we found that colon cancer was more common in males (61 cases) than in females (59 cases) with a small gap difference similar to what was reported in the Saudi Cancer Registry in 2015, where the number of cases in males was slightly higher than females (475 and 420 cases, respectively) [15]. Moreover, Brenner et al. reported that the mean age of colon cancer in German male patients was 66 years, while females had a mean age of 42 years [16]. The mean age of male patients in our study is 61 years, which is relatively comparable the global age of occurrence, but perhaps more closely resembles the median age of diagnosis of 59 years in males (range: 8 and 94 years) and 57 years in females (range: 15 and 102 years) as per the Saudi Cancer Registry [15]. 

Colon cancer is associated with modifiable and unmodifiable risk factors. Several modifiable risk factors have an established association with the increasing occurrence of colon cancer. For example, growing evidence indicates that obesity is a crucial risk factor for cancer, especially for colon cancer. The association between body mass index (BMI) as an indicator of excess body weight and the risk of colon cancer has been investigated in several meta-analyses. One systematic review reported that individuals with higher obesity had a 22% higher risk of colon cancer as compared to individuals with stable weight. Moreover, the study found that every 5 kg increase in body weight was associated with a 4% higher risk of colon cancer [17]. Similarly, our results concluded that 14.1% of colon cancer patients had a high BMI and were categorized as obese. Although 17 patients were found to have the risk factor of obesity in our study, the existence of other risk factors cannot be excluded. 

A range of modifiable lifestyle factors increases the likelihood of developing colon cancer. A systematic review reported that smoking has been associated with both rectal and colon cancer with the former being at a higher risk [18]. Our study reported that 10 patients (8.3%) were smokers at the time of diagnosis which emphasizes the association between colon cancer and cigarette smoking. We also found that the most common site of colon cancer was the left side with a total of 66 patients (55%), 50 of which had cancer in the sigmoid colon, and 16 had cancer in the descending colon. Similarly, Golfam et al. investigated colon cancer in 218 patients and found that 50 patients (23%) suffered from left-sided colon cancer, while 26 patients (12%) suffered from cancer on the right side [19]. 

The main treatment modality for localized non-metastatic stage colon cancer is surgical resection. Surgery alone for patients with Stage I colon cancer can be curative, and there is no role for adjuvant chemotherapy for them. Patients with low-risk Stage II colon cancer, which comprised the majority of our cases (32.5%), can be on surveillance without adjuvant therapy with an excellent prognosis. Current National Comprehensive Cancer Network (NCCN) and American Society of Clinical Oncology (ASCO) guidelines strongly recommend adjuvant therapy for selected Stage II colon cancer with high-risk and all Stage III [9, 20].

One study showed improved overall survival in Stage II and III colon cancer patients who underwent surgery followed by chemotherapy [21]. In our study, adjuvant chemotherapy (64 patients, 53.3%) encompassed the majority of cases, while 56 patients (46.7%) underwent surgery alone. Nevertheless, the recurrence rate in patients who underwent surgical resection with adjuvant chemotherapy (n = 10; 15.6%) was slightly lower than in patients who underwent surgery alone (n = 12; 21.4%). A large retrospective cohort of 818 patients analyzed the recurrence percentage of colon cancer treated with surgery alone and found that 40% of cases had colon cancer relapse, signifying the preferable outcome associated with adjuvant chemotherapy modality [22].

There were several limitations in our study, such as small sample size and the lack of survival data, for the period 2009 - 2018.

## Conclusions

In Saudi Arabia, colon cancer is one of the most diagnosed cancers with an increasing incidence over the last decade. Surgery remains the mainstay of treatment but, alone, may not decrease the recurrence rate, as demonstrated in this study. Adjuvant chemotherapy and surgical resection constitute the paramount treatment modalities of colon cancer. However, these treatment modalities need further understanding as to whether other factors, such as stage, histological type, and age, play a role in the recurrence rate. In conclusion, our results indicate that patients who underwent surgery alone had a higher recurrence rate in comparison to patients who underwent adjuvant chemotherapy. It is critically necessary to further demonstrate the effect of colon cancer treatment modalities on the recurrence rate in a larger population.
